# A recurrent epidermoid cyst of the spleen: report of a case and literature review

**DOI:** 10.1186/s12957-016-0857-x

**Published:** 2016-04-01

**Authors:** Pasquale Cianci, Nicola Tartaglia, Amedeo Altamura, Alberto Fersini, Fernanda Vovola, Francesca Sanguedolce, Antonio Ambrosi, Vincenzo Neri

**Affiliations:** Department of Medical and Surgical Sciences, University of Foggia, Luigi Pinto str 1, 71122 Foggia, Italy; Department of Clinical and Experimental Medicine, University of Foggia, 71122 Foggia, Italy

**Keywords:** Splenic cysts, Epidermoid cyst, Laparoscopic surgery, Immunohistochemistry, Carbohydrate antigen 19-9

## Abstract

**Background:**

Splenic cysts are rare disease. Epidermoid cysts of the spleen belong to the primary nonparasitic splenic cysts group. They are an unusual event in surgical practice. Usually, epidermoid cysts occur in children and young female. Most often, they are asymptomatic, but they may present with abdominal discomfort.

**Case presentation:**

We are reporting a rare case of a 23-year-old female came to our attention with history of intermittent pain and previously undergone two times to laparoscopic decapsulation of the cyst in others institutions. During hospitalization, serum and intracystic levels of tumor marker CA19-9 increased. Enhanced CT of the abdomen showed recurrent large cyst in the upper pole of the spleen with satellite nodules. Laparotomic total splenectomy was performed. Histopathological and immunoreactive examinations were executed, and they revealed stratified squamous epithelium on the inner surface of cystic wall, which was positive for EMA, CEA, and CA19-9. The diagnosis of epidermoid cyst was confirmed.

**Conclusions:**

Recently, the surgical approach is changing towards conservative treatments in order to save the spleen in young patients for immunological reasons. Sometimes, this target is not achievable. In such circumstances, like recurrent large cyst, anomalous anatomical relationship to the surrounding tissues, total splenectomy is safe and necessary.

## Background

Epidermoid cysts of the spleen are very rare. They are mainly primary nonparasitic splenic cysts (PNSC), and constitute approximately 10 % of the total cysts [1–4]. These lesions occur most commonly in children and young female patients, and carry a good prognosis [2–5]. Since splenic cysts are rarely symptomatic, and slightly less than 30 % are asymptomatic [6], they are detected in most cases by incidental imaging studies. The most common symptoms are nonspecific such as left upper abdominal mass, followed by left upper abdominal pain and epigastric pain. Few patients may present with complication, such as infection, rupture, and bleeding. Recently, the rate of newly diagnosed cases is increased due to the latest developments in diagnostic techniques, nevertheless only a few cases have been reported in literature so far [7]. Traditionally, a surgical treatment is recommended for symptomatic cysts larger than 5 cm [8]. We present here an unusual case of a recurrent epidermoid cyst of the spleen.

## Case presentation

**Fig. 1:**
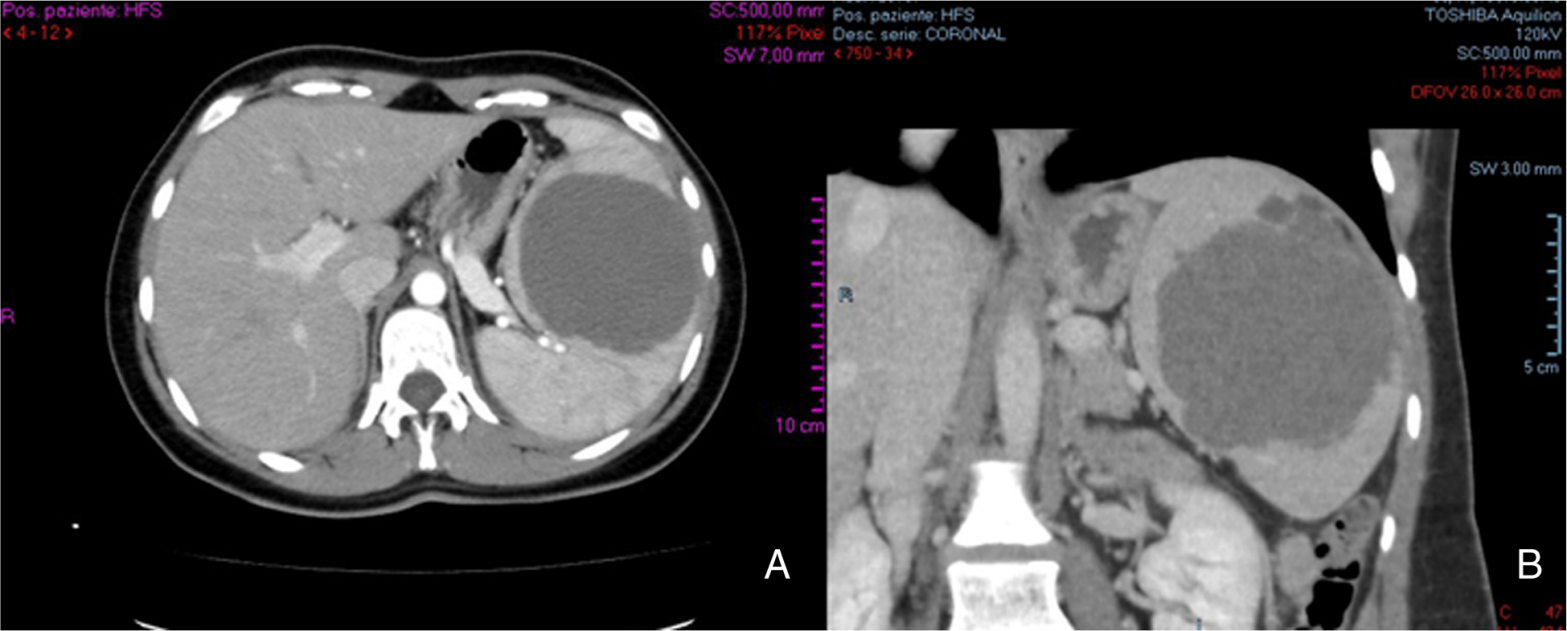
Preoperative contrast-enhanced abdominal CT scan: **a** the axial projection shows the recurrent splenic mass (7.7 × 8.5 cm), disclosing absence of calcifications, no contrast uptake; **b** the sagittal projection shows the presence of satellite nodules, and the splenic parenchyma almost totally replace

**Fig. 2:**
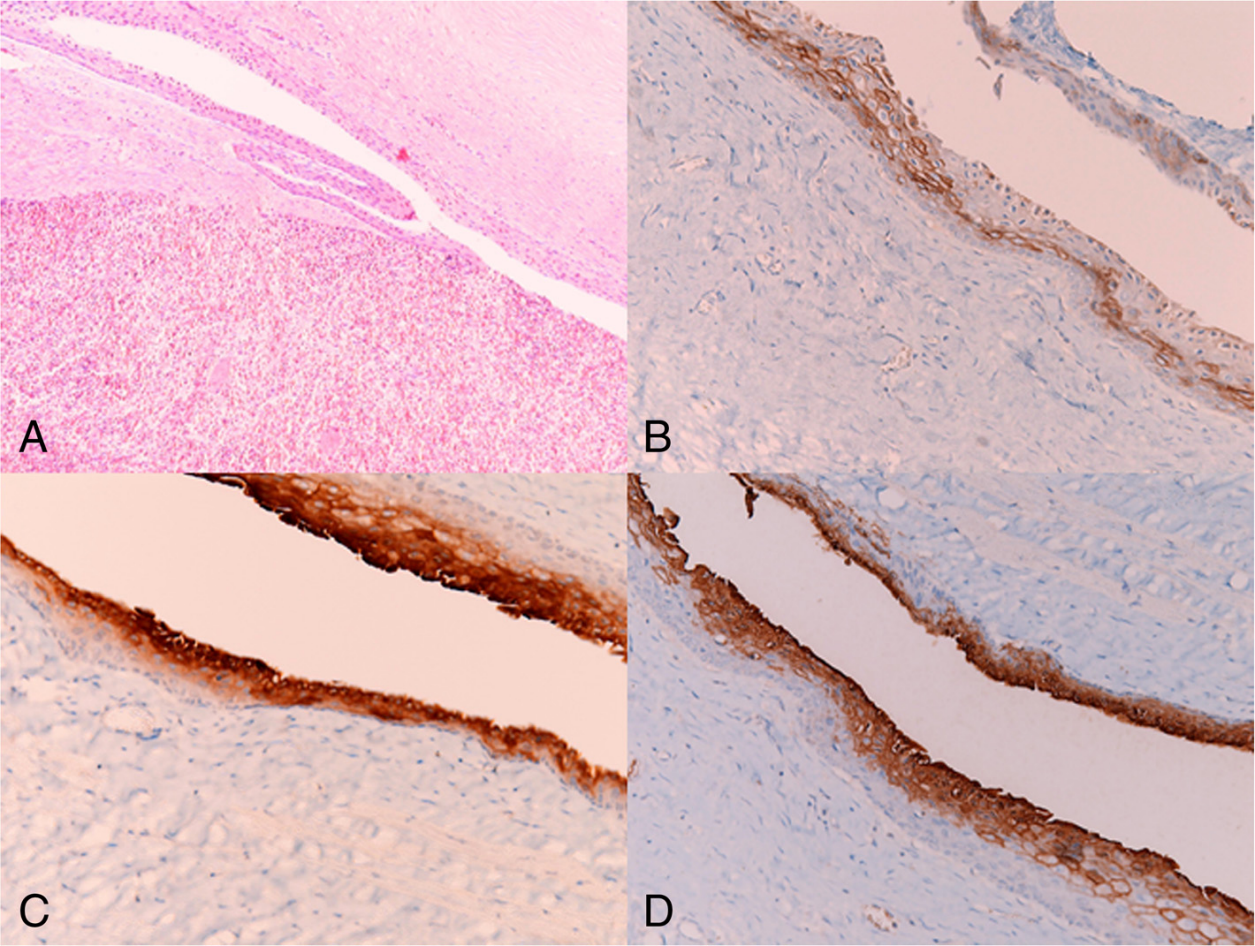
Histological examination shows **a** an epidermoid cyst with fibrous walls and a stratified squamous lining (*top*) within the spleen parenchyma (*bottom*) (hematoxylin and eosin, original magnification ×100); **b** the epithelium is immunoreactive with EMA, **c** CEA, and **d** CA19-9 (original magnification ×200)

A 23-year-old female was admitted to our department with dull pain and feeling of weight in the left upper abdomen which increases in the upright and prone position and after meals. Her medical history began about 2 years ago when she was admitted to a different surgical Department with the same symptoms; the CT scan revealed a large well-defined cystic mass of 15 × 12 cm in size in the upper pole of the spleen. Elective laparoscopic decapsulation was performed along with puncture of the cyst in order to reduce its size. At histological examination, the cyst was composed of a thick fibrous tissue wall lined by a stratified squamous epithelium. After 5 months of follow-up, a CT scan demonstrated a recurrent cystic lesion; subsequently, the patient turned to another surgical team who performed further laparoscopic decapsulation with drainage of the lesion. In this case, the microscopic examination of the removed wall fragment showed a coating of squamous epithelial cells. Eight months later, the patient complained again of pain and sense of weight in the same location, and a CT examination revealed the second recurrence of the cyst. She came to our attention 10 months later. On the clinical examination, pre-existing surgical scars were noted, and deep palpation of the left hypochondrium evoked an intense pain. After preliminary tests, an abdominal CT scan with intravenous contrast was performed that confirmed the recurrent splenic mass (7.7 × 8.5 cm), disclosing absence of calcifications, no contrast uptake, no involvement of splenic vessels, and the presence of satellite nodules, without compression of the spleen, pancreas, and left kidney (Fig. 1). Serological examinations allowed us to rule out parasitic and viral etiology (Echinococcus granulosus, Epstein Barr Virus, Human Herpes Virus 1/2, Cytomegalovirus), and showed slight anemia (HGB 10.80 g/dL, HCT 32.40 %, MCV 62.50 fl) and a normal white blood cell count (5.7 × 10^3^/uL). For the first time, compared to the previous blood findings, the level of the CA19-9 was raised to 54 UI/ml (normal range 0–37 UI/ml). In agreement with the hematologists, we decided to undergo the patient to open splenectomy (elective laparotomy and splenectomy) due to the size and site of the cyst, after a vaccine prophylaxis of 1-month duration with administration of Pneumo23, Mencevax, and Acthib. Macroscopically, the fully resected spleen weighed 770 g, with dimension of 18 × 13 × 7 cm, and was almost totally replaced by the large cyst. Histological examination disclosed a large multilocular cyst with fibrous walls lined on their inner surface by squamous epithelial cells with no nuclear atypia, which were immunohistochemically positive for epithelial membrane antigen (EMA), carcinoembryonic antigen (CEA), and CA19-9 (Fig. 2). The final pathological diagnosis was an epidermoid cyst of the spleen. No perioperative and postoperative complications occurred. The patient was discharged on the fifth postoperative day with all the blood parameters within the normal range, including CA19-9.

## Conclusions

Splenic cysts are rare diseases that are diagnosed incidentally during imaging studies or in everyday surgical practice. This is due to the lack of typical clinical presentation, since they are often asymptomatic; the incidence rate is approximately 0.07 %, as reported in a large case series of about 42,327 autopsies [9, 10]. They usually occur in females and can be seen more commonly in individuals under 40 years of age [2]. The first case was reported in 1929 by Andral and since then, many pathophysiological hypotheses have been proposed, the main one being infolding or entrapment of peritoneal mesothelial cells in the splenic parenchyma during embryogenesis in the intrauterine life (mesothelial invagination theory, endodermal inclusion theory) [11, 12]. Splenic cysts are classified worldwide according to Martin’s criteria [4]: type 1 are true cysts with a lining epithelium, while type 2 are false cysts without a defined lining epithelium. The first group includes both nonparasitic and parasitic cysts, the latter ones being most frequently caused by Echinococcus granulosus [13, 14]. Type 1 nonparasitic cysts have been further divided into congenital (epidermoid, dermoid) and neoplastic (hemangiomas, lymphangiomas) cysts, among which hemangiomas and dermoid cysts are the most and least common, respectively. The second group includes the so-called pseudo-cysts which are usually related to trauma, hemorrhage, abscess, and infarction [10], and occur much more frequently than epithelium-lined true cyst [15, 16]. Primary splenic cysts constitute 10 % of all nonparasitic cysts and can be found in the regular spleen or in the intrapancreatic accessory spleen [17]. There are some differences regarding demographics and clinical presentations of epidermoid cysts between the spleen and intrapancreatic accessory spleen. Epidermoid cyst tends to occur more frequently in the group of the regular spleen and the average age is lower than intrapancreatic accessory group. Most of epidermoid cysts of the spleen are much larger than intrapancreatic accessory spleen ones [6]. Generally, the tumor size correlates with clinical presentation and small cysts are symptomless, but large lesions of the spleen tend to present symptoms related to bulky size, such as left upper abdominal pain, upper abdominal mass, and epigastric pain and fullness. Few patients may present thrombocytopenia [18]. Our case, in fact, presented the most common clinical finding: left upper abdominal pain, and hematological and biochemical investigations were within normal limits except CA19-9. Accurate clinical diagnosis of primary epithelial cysts is difficult. The differential diagnoses for a cyst in the spleen include parasitic echinococcal disease, congenital cyst, postraumatic pseudocysts, infraction, infection, pyogenic splenic abscess, metastatic disease, and cystic neoplasm. The instrumental investigations are important to complete the diagnosis. It is possible to employ ultrasonography (USG), magnetic resonance (MR), and computerized tomography (CT). USG could differentiate solid and cystic lesions, identify calcifications, and recognize some intra-cystic septa or irregular walls. On T2-weighted MR images, the cyst is hyperintense, with intensity of signal equal to water. Generally, on T1, signal is hypointense but it can be increased according to the contents of cyst, for example, in case of a hemorrhagic type [19]. We used CT scan to complete the diagnosis and to have information in regard to the morphology of the cyst, the nature of fluid and the exact location and its relationship with adjacent structures. At the same time, we affirm that radiologically distinguishing between true and false splenic cysts not seem to be certain preoperatively and the final diagnosis relies on the histopathological examinations. They reveal that epidermoid cysts have stratified squamous epithelium with a fibrocollagenous cyst wall, as well as in our case. For epidermoid cysts located in the spleen, surgical choices include total or partial splenectomy and the treatment is recommended when the cyst is symptomatic or malignancy is suspected, and in order to prevent serious complications such as rupture, bleeding, and infection of the cyst [11, 20]. These procedures can be achieved by either traditional open method or laparoscopic approach. Recognizing the role of the spleen in immunologic function, splenic preservation is a legitimate concern, especially in very young patients [21]. Various alternative approaches aimed at avoiding splenic tissue destruction have been tried. They include aspiration alone, aspiration and sclerotherapy, percutaneous drainage, incision and drainage, and decapsulation of the cyst [15, 16]. All of these treatments can be considered more effective for cysts smaller than 5 cm than those of large dimensions. If the surgeon decides to perform conservation treatments for masses larger than 5 cm, the risk of recurrence increases [8]. About us, for the management of epidermoid splenic cysts, it should consider three important parameters: the size of the lesion, anatomical relationship with splenic parenchyma and surrounding organs, and age of the patient. Even if the surgeon should avoid removing the spleen in children and young patients, any type of conservative surgical treatment modality has little value in cases such as a very large cyst more than 5 cm, with dense vascular adhesions to the surrounding tissues and structures, and multiple cysts. In these situations, we recommend total splenectomy.

## Consent

Written informed consent was obtained from the patient for publication of this Case report and any accompanying images. A copy of the written consent is available for review by the Editor-in-Chief of this journal.

## Abbreviations

CA19-9: carbohydrate antigen 19-9
CEA: carcinoembryonic antigen
CT: computed tomography
EMA: epithelial membrane antigen
MR: magnetic resonance
PNSC: primary non-parasitic splenic cysts
USG: ultrasonography
